# Social Epidemiology and Global Mental Health: Expanding the Evidence from High-Income to Low- and Middle-Income Countries

**DOI:** 10.1007/s40471-017-0107-y

**Published:** 2017-04-18

**Authors:** Joanna Maselko

**Affiliations:** Department of Epidemiology, Gillings School of Global Public Health, University of North Carolina at Chapel Hill, 2105e McGavran-Greenberg Hall, Campus Box 7435, Chapel Hill, NC 27599-7435 USA

**Keywords:** Global mental health, Social epidemiology, Health disparities, Low- and middle-income country (LMIC), Common mental disorders, Socioeconomic status

## Abstract

**Purpose of the Review:**

The vast majority of research on the social determinants of mental health has been generated from high-income country (HIC) populations, even as the greatest health disparities, and greatest disease burden, is observed in lower- and middle-income countries (LMICs). The goal of this review is to examine the evidence base on how key social epidemiology constructs relate to mental health in LMIC contexts. A special focus is on points of departure from the HIC knowledge base, gaps in overall understanding, and opportunities for social epidemiology to make a significant contribution.

**Recent Findings:**

A growing body of literature suggests that there is significant heterogeneity, both in the direction and magnitude, of association between factors such as socioeconomic status, income inequality, gender, and social networks/supports and mental health in LMIC. For example, higher levels of education and being married can be risk factors for worse mental health among women in certain contexts. However, many studies have methodological limitations that make causal inference difficult. Poverty alleviation interventions offer a unique opportunity to examine the impact of improving economic resources and mental health.

**Summary:**

Much remains unknown about the impact of key social factors on mental health in LMIC. Findings from HICs may not apply to LMIC populations, since the meaning and distribution of a given social variable may differ significantly from what is commonly observed in HICs. These points of departure point to opportunities for social epidemiology to make a contribution to the field of global mental health.

## Introduction

Over the last decade, health researchers and funders have become increasingly concerned with the 10/90 gap: the phenomenon that only 10% of health-related research addresses problems of 90% of the world’s population [1, 2]. This inequality is of special concern to social epidemiology given the implication that the vast majority of research applies to the wealthiest and healthiest countries, with the vast health disparities occurring in the remaining 90% of the global population. The topic of health disparities in low- and middle-income countries (LMICs) has emerged as an active area of research and provides an opportunity for social epidemiology to meaningfully contribute to research on key social determinants of health beyond high-income country (HIC) contexts [3•, 4–6].

The shift from social determinants as they manifest in the USA, UK, and other European countries to their manifestation in LMIC contexts is not, however, completely straightforward. In many LMICs, the meaning and distribution of a social factor, such as income inequality or education, may depart drastically from what is commonly observed in HICs. For example, in South Africa, the income inequality measure, the GINI index, is 63 (in 2011) and a very small middle class, while in the USA, the GINI index is 41 (in 2013) with a much larger middle class, making the USA a much more equitable country [7]. In the South African context, the independent effect of income inequality on health is more difficult to disentangle from the effects of poverty with which it is entangled. Focusing on education, in Bhutan, 68% of the population 25 years and older has received no schooling, and this number represents a 20% gender difference (57.8% of men and 77.8% of women have had no schooling). The comparable statistic in the USA is 0.4%, with no detectible gender difference [8]. So, while in HIC such as the USA, much of the discourse is about disparities in high school graduation rates and the value of a college education, the health benefits of the first few years of primary education are not nearly as well characterized. And yet, this is the margin at which education affects the health of a much larger portion of the world’s population. Such differences between HIC and LMIC emerge with most social determinants.

The field of global mental health (GMH) is explicitly concerned with reducing disparities in mental ill-health and hence is very concerned with social determinants, making it a natural complement to social epidemiology. A key goal of GMH has been to reduce disparities between and within countries through increasing access to culturally appropriate evidence-based interventions as well as addressing social determinants of mental ill-health [9–11]. Mental ill-health is broadly defined, and the vast majority of research focuses on depression and anxiety, often referred to as the common mental disorder (CMD), in addition to a growing body of research on schizophrenia, dementias, autism, substance use disorders, and self-harm [12–15]. Together, the broader category of mental disorders account for up to 13% of disability adjusted life years (DALYs), making them the second leading cause of the global burden of disease after cardiovascular diseases [16].

Drawing on both social epidemiology and global mental health, the goal of this article is to examine how key social determinants, such as socioeconomic status, income inequality, or gender, relate to mental health in LMIC contexts. A main focus is on points of departure from the HIC knowledge base, gaps in overall understanding, and opportunities for social epidemiology to make a significant contribution.

### Social Determinants Within Global Mental Health

The social context has always been important in global mental health, both in shaping the risk of a neuropsychiatric disorder as well as its likelihood of recovery [9, 10, 14, 17, 18]. The importance of social determinants within global mental health can be traced to the fields’ anthropological roots and the cross-cultural psychiatrists who first described the experiences of mental illness outside of Western, HICs [19, 20]. Approaching mental health with an inherently social, anthropological lens, an understanding of mental ill-health emerged that is embedded in one’s social environment and often precipitated by factors such as poverty or gender inequality [4, 21, 22]. Although the anthropological approach has sometimes been in tension with the more medical or public health approach to mental health, the importance of social determinants has rarely been questioned [23].

However, the fact that social determinants are acknowledged, or even accepted, does not mean they are well understood. There is significant heterogeneity in the findings linking key social factors to mental health [24] and tensions have risen about which factors are ‘really’ important for mental health vs. just being markers for something else [25]. This lack of clarity has led to calls for more, higher quality, research into the social determinants of mental health in LMICs [3•, 5]. Specifically, much more knowledge is needed in low-resource settings to (1) better understand the causal relationships between social factors and diverse mental health problems, and (2) elucidate the mechanisms through which social factors impact mental health. Such questions are also of central interest to social epidemiology.

### Socioeconomic Status and Poverty

Of all the social determinants of mental health, socioeconomic status (SES) has received the most attention [3•, 24, 26]. Although a full review is beyond the scope of this paper, a majority of studies show an inverse association between lower SES and numerous mental health outcomes [24, 26]. However, there is significant heterogeneity in findings, and this might be due in part to lack of clarity of both conceptual and methodological issues [27•]. Two key areas of debate are measurement of SES and directionality of effects.

#### Measurement

The majority of studies examining SES and mental health fail to clearly define the choice of SES measure, or explain its theoretical rationale [27•]. Commonly used indicators include consumption/expenditure amounts, income, occupation, education, or assets, leading to seemingly conflicting findings. Because each of these measures captures a different aspect of SES, with potentially a different pathway linking it to mental health, homogenous findings should not be expected, within or between countries. It has been challenging to synthesize the diverse findings of multiple SES indicators in a coherent way.

Many studies of SES use the terms SES and poverty interchangeably without explicitly acknowledging how the choice of term influences analyses or interpretation of results [3•, 27•]. Dichotomous proxies for poverty are frequent, such as living on less than $2/day, being in debt, living in overcrowded conditions, or experiencing food insecurity. While a dichotomous poverty indicator is arguably easier to measure, fits well with the poverty alleviation agenda, and can be useful for policy makers, it oversimplifies the SES-mental health relationship. Importantly, this reliance on poverty, with comparisons of those with the highest levels of adversity with everyone else, obscures the gradient nature of the SES-mental health relationship. There is little reason to expect the gradient to be any less strong in LMIC contexts than in HIC ones. The focus on high-risk poverty can also lead to an unintended consequence of weakening the case for improving access to prevention and treatment options because of a belief that mental health problems are ‘inevitable’ at such high levels of adversity. Such oversimplifications can be prevented with a more fine-grained measure of SES which, in turn, facilitates a better understanding of the nature of the association between socioeconomic status and mental health at all levels of adversity. To guide future work on SES and mental health, researchers have recently proposed a set of guidelines for choosing an SES measure, including (a) choosing a measure based on a theoretical basis with conceptual and contextual relevance, (b) determining and stating the unit of measurement and time period, and (c) being clear about how categories and groupings were created and why [27•]. To guide an improved theoretical basis for measurement, researchers would benefit greatly from the extremely rich body of scholarship that examines the theoretical underpinnings and lived experience of poverty and social class (e.g., [28–31]).

#### Directionality

The idea of reciprocal and cyclical relationship between poverty and mental health is mostly accepted within global mental health [17]. The term ‘social drift’ is used more often than ‘social selection’ since this portion of the cycle, where a person with mental illness becomes poor as a result of their illness, is also understood to be shaped by social factors related to family and community support and integration. It has been hypothesized that social drift may be a stronger contributor to the SES gradient observed with disorders such as schizophrenia, while social causation is more likely to explain the depression SES gradient [5]. The relative magnitude of effect in both parts of the cycle has direct implications for allocation of resources whose goal is reducing disease burden. Is an anti-poverty measure or an increase in access to mental health care a ‘better’ use of resources? In areas where poverty is overwhelming, efforts at treating mental illness without addressing adversity can seem naïve at best, and an encroachment of Western biomedicine and pharmaceutical industries at worst [32]. On the other hand, changing more upstream determinants is extremely difficult, and usually outside of the expertise of those working in global mental health. This dichotomized view of social vs. biomedical approaches has the risk of becoming polarized even as it is ultimately overly simplistic and unproductive [18, 23, 33]. The degree to which social determinants such as SES impact risk of mental health problems, which in turn further reinforce SES vulnerabilities is an empirical question that social epidemiology is well poised to address.

Unlike in HIC where experimental data on social determinants and mental health is fairly scarce, in LMIC, some of the most informative empirical contributions are from poverty alleviation interventions that assess mental health outcomes, as well as mental health interventions that assess socioeconomic outcomes [17, 26]. These interventions provide evidence on how a change in economic circumstances can impact mental health, and whether improvements in mental health can lead to improved economic outcomes. Additionally, they provide information on the extent to which mental health problems may get in the way of people taking full advantage of available socioeconomic opportunities. In other words, they help point to how the cycle of poverty-mental health problems may be broken [5, 17]. Here, I will focus on the social causation part of the cycle with economic interventions and their impacts on mental health.

Loan and micro-enterprise interventions provide access to credit. The goal of these interventions is usually to provide enough capital (either through a loan or grant) so that the participant can invest those funds in a small business enterprise or other income generating activity (e.g., purchase a plot of land that can be farmed or raw materials to make textiles to be sold). The findings from these interventions are mixed for mental health outcomes, even as financial indicators show some improvement. For example, a trial in the Democratic Republic of Congo helped with saving and small loans found improvements in per capita food spending but not any mental health symptoms [34]. The authors hypothesized that the increased savings may not have been sufficient to improve mental health in this traumatized population; a combination of mental health and economic support may have been needed. Another microenterprise intervention in Uganda reported similar results: monthly cash earnings increased 16 months after start of the program, but there was no impact on depression symptoms [35]. These, and other similar interventions, are complex, with multiple components aimed to help participants lift themselves out of poverty. One might argue that in order to succeed, they require a level of functioning from the participants that might be too high. However, findings with simpler interventions also vary. For example, a program in South Africa was based solely on improving access to loans with existing banks, reported overall increased levels of stress 6–12 months post intervention, while depression symptoms reduced among men, but not among women in the study [36]. The mixed findings may result from several factors. It is possible that mental health impacts take longer to emerge, once not only the immediate economic circumstances improve but a more long-term security is achieved. Alternatively, the magnitude of the economic boost resulting from the intervention may not be sufficient given the very low starting SES of the participants. Finally, the mechanism of supporting entrepreneurial activities may only benefit a portion of participants—those who have the skills and aptitude for a small business pursuit. This would lead to heterogeneous impacts on both economic and mental health outcomes.

Another group of poverty alleviation programs that address these questions are cash transfer interventions, whether conditional on activities such as sending children to school and getting immunized, or unconditional, no-strings attached, cash transfer programs. Mexico’s well-documented *Oportunidades* program was an intervention where women received a significant stipend, conditional on several health-related behaviors including prenatal care, immunizations, and educational workshops [37]. The stipend was roughly 25% of household income and, at the time of the evaluation, women had been in the program between 3.5 and 5 years. Depression symptoms were significantly lower in the intervention group and this effect was partially mediated through both a reduction in stress and increase in perceived control [38•]. It is noteworthy that the positive impact was greater among women who were relatively better-off at the start of the study; there is some debate about whether interventions that promote health overall may increase inequalities because those who are at the very bottom are not able to fully take advantage of a program’s benefits [39]. The long-term evaluation, at 5 years, is also notable when compared to the previously described interventions that were evaluated within a year or two of the program rollout. It might take time for mental health effects to become apparent. While the *Oportunidades* program is one of the best documented, other similar cash infusion programs at a smaller scale, such as significant wage increases at factories, similarly report lower depressive symptoms among program beneficiaries [40].

In summary, poverty alleviation interventions provide important insights into the SES-mental health relationship: We have learned that purely economic interventions among poor participants may not have enough impact when other significant sources of distress remain. Individuals may also need a broader safety net than one provided by cash and/or the amount of money needed may be much more significant than previously assumed [34, 35]. The evaluation of poverty reduction programs from the perspective of mental health is thus a very powerful approach toward disentangling the mechanisms through which SES impacts population mental health [26].

### Income Inequality

Discussions of poverty often dovetail with those about income inequality [3•] and a few studies have attempted to quantify the impact of income inequality on mental health in LMIC. As mentioned above, levels of income inequality are generally significantly higher in LMIC than in HIC and LMICs tend to have a much smaller middle class. These countries simultaneously have high poverty and inequality rates, making it more difficult to isolate the impact of inequality per se. As might be expected, findings have been mixed. Several studies from South Africa and Mexico found no association [41, 42], while a study from Sao Paulo Brazil found income inequality to be positively associated with depression [43]. Several reasons may explain these findings. One is methodological: studies utilizing larger geographical measures of inequality, such as district or state levels, seem more likely have null results (South Africa, Mexico), while more micro level measures are more likely to find an association (Brazil). Other potential explanations include the fact that, in high inequality LMICs, there may not be enough variation in inequality, especially if a ceiling effect has been reached; the GINI coefficient may not be the appropriate indicator; or the common focus on depression may miss other mental health problems susceptible to the effects of inequality, such as substance use [41–44]. In addition to these challenges, Adjaye-Gbewonyo hypothesized potential contextual reasons for why an association might not be observed in a country such as South Africa: people in South Africa may be more tolerant of high-income inequality. This could be either because they consider income inequality as temporary on the way to improved post-apartheid economic development. Alternatively, in other contexts, people’s expectations for equality may be lower, and communities so segregated that people are less aware of inequality while being more impacted by their own SES level. It has also been suggested that income inequality is only important for health in places of low poverty levels such as in HIC, while in places with high poverty levels, health is driven by absolute poverty [45]. Much remains unknown about how income inequality interacts with other social, cultural, and economic factors to influence mental health [3•].

### Gender

After SES and poverty, gender is one of the most well-described social determinant of mental health in LMIC. Gender differences in CMD prevalence are regularly reported and gender is routinely included in analyses predicting mental health outcomes. As in HIC, in LMIC women are at higher risk of CMD and almost all studies acknowledge women’s vulnerability resulting from their lower status in society as a key driver of higher CMD rates [14, 46, 47]. It is not clear to what extent the excess risk associated with female gender for CMD differs in magnitude between LMIC and HIC. The gender difference in alcohol use disorders does appear to be larger in magnitude in some LMICs, with men’s risk up to 20 times greater than what is observed among women [14]. This difference is potentially driven by lower prevalence of women consuming any alcohol in many LMICs in combination with a much greater proportion of men who consume any alcohol, consuming it at hazardous levels. Moderate alcohol consumption appears to be more normative in HIC.

Given that gender interacts with social institutions such as marriage or the labor market, we may expect different epidemiological patterns of mental health problems among women than those observed in HIC, Western countries [48]. For example, in India, higher education has been associated with a twofold *greater* risk of suicide among women [49]. Several hypotheses have emerged about why a higher educational attainment might be a risk factor. In certain communities, efforts to encourage girls’ education have resulted in young women being, on average, more educated than young men [50]. These years of education sometimes come with significant personal sacrifice and anthropological work has shed light on the difficulties and stressors faced by some women as they try to achieve their education goals; stressors that, in turn, may lead to mental health problems [51]. However, even once education is complete, a higher level of education may not translate into economic empowerment, as women often cannot successfully compete with men for scarce jobs. As women become married, they may revert to traditional gender roles and a lower status, which can be all the more distressing after having been ‘temporarily empowered’ through their education. The lower status of newly married women might also help explain why widowed or divorced women can be at *lesser* risk for suicide and CMD compared to those who are married [14, 49]. With a strong emphasis on childbearing, there is evidence that adverse life events related to reproduction may be partially responsible for the high burden of mental health problems among women in places like India [46]. Interpersonal violence may be another potential mechanism linking negative reproductive outcomes with CMD [52].

### Social Networks and Supports

Although access to social support networks, especially during times of adversity, is consistently shown to have a protective effect on mental health [53–55], there are several notable differences between the HIC and LMIC bodies of research.

One key difference is the greater importance given to family-based networks. This is, in part, a result of a higher prevalence of extended, multi-generational, family households in LMICs; an arrangement that encourages a higher level of caregiving expectations and support between family members. Consistent with a focus on childbearing mentioned above, the majority of the research in this area has focused on maternal mental health, especially the perinatal period. For example, studies in South Asia have found that the presence of the grandmother in the household has generally been correlated with better mental health outcomes among mothers and children, with the most likely pathway being through provided social support [56, 57]. One commonly described example of this family-based social support is the practice of ‘confinement’ or ‘chilla’ of a new mother during the first 30–40 days after birth. The specifics of this period vary cross-culturally but in almost all circumstances, it is a period for rest and bonding between the mother and her new infant. The mother is relieved from household chores and generally stays protected indoors. This practice has been found to be protective against maternal depression [11, 58] and is understood to indicate a strong social support, family-based, network. Indeed, it is only possible with a commitment from multiple people to provide significant support.

However, other research has revealed some of the complexities around activating one’s social support network. One’s social network can be a source of shame or stigma, as was the case in one study where food-insecure mothers feared asking for help due to the shame they felt for not being able provide food to their family [59]. This fear of revealing a perceived weakness may apply both to close members of one’s networks, such as other family members, or those who are less close, such as neighbors. The worry about stigma coming from one’s network can also hinder participation in other poverty alleviation type interventions as well [60]. It is plausible that the impact of both positive and negative aspects of social networks, e.g., support vs. shame, might be stronger in cultures that are more community vs. individual focused, although this hypothesis remains to be tested.

Much remains unknown about other aspects of social networks, such as men’s networks, more extended non-kin-based networks, or how technology facilitates social network contacts. For example, cellular phone usage has been found to be associated with higher stress in HIC, but a recent study in Uganda found that cell phone ownership and usage was associated with better mental health, especially among those whose families did not live nearby [61]. In locations with high migrant, or displaced, populations, the improved social connectivity from internet or cellular technology may be especially protective. Such differences in the structure of social networks, how they are activated, and the supports they provide are likely to continue to emerge with continued study.

### Conclusions and Going Forward

This targeted review reveals that much remains unknown about how social determinants shape the risk of mental ill-health in LMIC. The examples of SES, income inequality, gender, and social networks/supports suggest that, while some knowledge from HIC does translate to LMIC, a considerable body of evidence does not. Importantly, it is not always clear a priori which lessons transfer well and which ones need significant revisions. These same challenges apply to social factors such as race/ethnicity, neighborhood effects, or social capital that were not discussed in this review but are also areas of active research (e.g., [62–64]). The intersection of global mental health and social epidemiology offers an exciting opportunity to address these challenges and advance the knowledge base of social determinants of mental health in LMIC.

Cross-disciplinary collaborations are key in this effort and the fields of economics and anthropology will continue to play a significant role in GMH. For example, development economists are increasingly aware of the importance of mental health in poverty alleviation efforts: mental health problems, such as depression or anxiety symptoms, impact decision making and risk taking, which may in turn, affect how individuals respond to economic changes [65, 66]. A recent call for the use of Amartya Sen’s Capabilities Approach to help inform how the global mental health agenda links with the economic development agenda is very promising [28, 67, 68]. The Capabilities Approach emphasis on upstream, structural factors that may limit individuals’ ability to function and achieve their goals is a useful theoretical framework to help guide social epidemiological research in this area. Anthropological work complements an economic or epidemiologic approach and helps us understand why there might be differences in how social factors impact health outcomes between different populations (e.g., [69]).

There is an urgent need for high quality, rigorous research with strong causal inference. Assessing the mental health impacts of poverty alleviation interventions is one area where the epidemiological, economical, and anthropological lenses can each offer unique insights. These interventions enable researchers to potentially estimate the magnitude of impact on mental health of a very specific social factor (financial benefit of a given amount). A related question is to what extent do existing levels of mental health impact the efficacy of the intervention [10]. Such interventions offer a wealth of very useful data but may have somewhat limited generalizability because of the specifics of the program. Knowledge gained from these experiments can be complemented by natural experiments, such as regional changes in legal age of marriage laws or a significant increase in public school teacher pay, which are sometimes more common in LMIC than in HIC. Finally, measures of mental health are increasingly being included in large demographic household surveys, often together with detailed information on many key social and economic constructs. In sum, the intersection of global mental health and social epidemiology is an exciting area of research, one with significant potential to make a positive impact on the health of the world’s most disadvantaged populations.
